# Annexin A2 is regulated by ovarian cancer-peritoneal cell interactions and promotes metastasis

**DOI:** 10.18632/oncotarget.1122

**Published:** 2013-07-14

**Authors:** Noor A. Lokman, Alison SF. Elder, Miranda P. Ween, Carmen E. Pyragius, Peter Hoffmann, Martin K. Oehler, Carmela Ricciardelli

**Affiliations:** ^1^ Robinson Institute, Research Centre for Reproductive Health, School of Paedriatrics and Reproductive Health, University of Adelaide, Adelaide, SA, Australia; ^2^ Research Centre for Infectious Diseases, School of Molecular Biosciences, University of Adelaide, Adelaide, SA, Australia; ^3^ Adelaide Proteomics Centre, School of Molecular and Biomedical Science, University of Adelaide, Adelaide, SA, Australia; ^4^ Department of Gynaecological Oncology, Royal Adelaide Hospital, Adelaide, SA, Australia

## Abstract

Our recent research identified the protein annexin A2 to be regulated by ovarian cancer-peritoneal cell interactions. This study investigated the role of annexin A2 in ovarian cancer metastasis and its potential utility as a novel therapeutic target, using *in vitro* and *in vivo* ovarian cancer models. Annexin A2 expression was examined by qRT-PCR and western blotting in ovarian cancer cell lines and immunohistochemistry in serous ovarian carcinoma tissues. Annexin A2 siRNAs were used to evaluate the effects of annexin A2 suppression on ovarian cancer cell adhesion, motility, and invasion. Furthermore, annexin A2 neutralizing antibodies were used to examine the role of annexin A2 in tumor invasion and metastasis *in vivo* using a chick chorioallantoic membrane assay and an intraperitoneal xenograft mouse model. Strong annexin A2 immunostaining was observed in 90% (38/42) of the serous ovarian cancer cells and was significantly increased in the cancer-associated stroma compared to non-malignant ovarian tissues. Annexin A2 siRNA significantly inhibited the motility and invasion of serous ovarian cancer cells and adhesion to the peritoneal cells. Annexin A2 neutralizing antibodies significantly inhibited OV-90 cell motility and invasion *in vitro* and *in vivo* using the chick chorioallantoic membrane assay. The growth of SKOV-3 cells and their peritoneal dissemination in nude mice was significantly inhibited by annexin A2 neutralizing antibodies. Annexin A2 plays a critical role in ovarian cancer metastasis and is therefore a potential novel therapeutic target against ovarian cancer.

## INTRODUCTION

Ovarian cancer is the most lethal gynecological cancer and ranks as the fifth most common cause of cancer-related death in women in the western world. It has been estimated that there will be 22,240 new cases of ovarian cancer and 14,030 deaths due to ovarian cancer in the United States in 2013 [1]. Despite improvements in the surgical treatment and the development of new chemotherapeutic agents over the last 10 years, ovarian cancer survival rates have not changed significantly. An increase of the ovarian cancer survival rate will require the successful development of more effective molecularly targeted therapies.

Ovarian cancer has a distinct predisposition for metastasizing via shedding of cancerous cells from the ovary into the peritoneal cavity and implanting onto the peritoneum that lines the pelvic organs. Once ovarian cancer cells adhere to the peritoneal cells, they migrate through the peritoneal layer and invade local organs. The local invasion of organs, such as the bowel, eventually results in the death of the patient.

Our group has recently explored the interactions between ovarian cancer-peritoneal cells using an *in vitro* co-culture system [2]. One of the proteins identified by 2D gel electrophoresis and mass spectrometry to be regulated by ovarian cancer-peritoneal cell interactions was annexin A2 [3].

Annexin A2 is a multifunctional calcium phospholipid binding protein which binds to collagen I, cathepsin B and tenascin-C [4], assists in maintaining the plasticity and rearrangement of the actin cytoskeleton [5] and a cellular redox regulatory protein [6]. Annexin A2 also plays an important role in the plasminogen activation system and acts as a tissue plasminogen activator (t-PA) receptor on the cell surface of endothelial and cancer cells, which mediates the conversion of plasminogen into plasmin [7, 8].

Various studies have found increased annexin A2 tissue levels in malignancies of the breast, pancreas, oropharynx, liver, kidney, and bowel (reviewed by [3]). Annexin A2 has been shown to promote cell invasion in malignancies of the breast, brain, liver, and pancreas [9-12] and enhances cell motility and cell adhesion of prostate and hepatocellular carcinoma cells [12, 13]. However, the knowledge on the role of annexin A2 in ovarian cancer is very limited. It was identified to be upregulated in ovarian cancer cell lines with high invasive capacity compared to those with low invasive properties [14]. Moreover, a large scale proteomic study identified annexin A2 to be upregulated in ovarian cancers when compared with normal ovarian tissue and benign lesions [15]. This study investigated annexin A2 expression in serous ovarian cancer tissues and cell lines and performed functional *in vitro* and *in vivo* studies to examine its role in ovarian cancer cell adhesion, motility, invasion and metastasis.

## RESULTS

### Expression of annexin A2 in human ovarian cancer tissues and peritoneal cells

**Figure 1:**
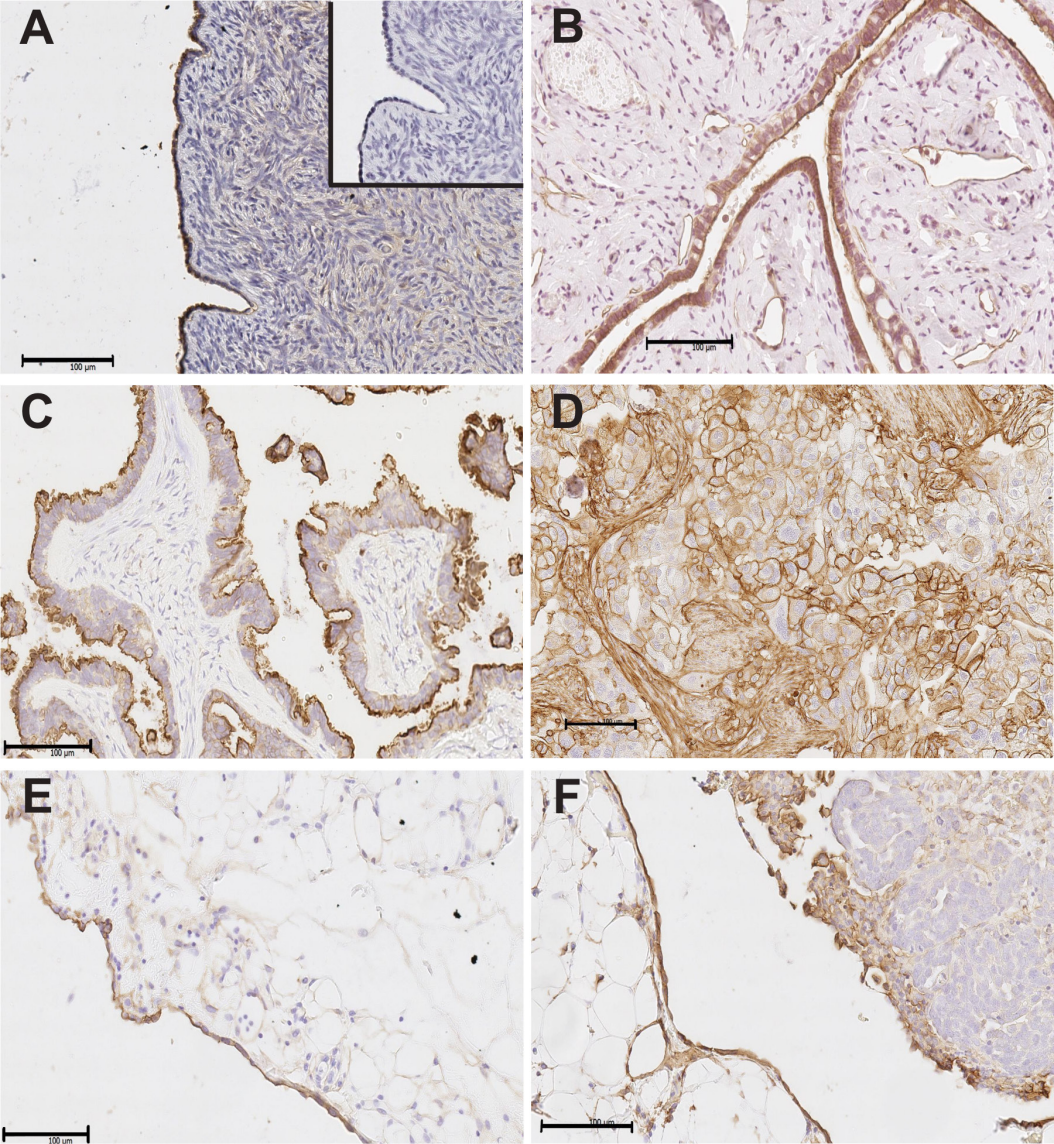
Annexin A2 immunostaining in human ovarian tissues and omental tissues Annexin A2 immunostaining is present in the epithelial cells of normal surface epithelium (A), serous cystadenomas (B) and serous borderline ovarian tumors (C). Annexin A2 immunostaining is present in membrane and cytoplasm of ovarian cancer cells and cancer associated stroma of stage 3 serous ovarian cancer tissues (D). Annexin A2 immunostaining is observed in peritoneal cells of the omentum (E) and serous ovarian cancer cells located adjacent to the peritoneal cells in omental implants (F). Insert in (A) is the negative control with no primary antibody. Magnification bar = 100 μm for all images.

Immunohistochemistry results showed positive immunostaining of annexin A2 in the epithelial cells of the normal surface epithelium (Fig. 1A), serous cystadenomas (Fig. 1B) and serous borderline ovarian tumors (Fig. 1C). In serous ovarian cancer cells, annexin A2 immunostaining was present predominantly in the membrane and cytoplasm but high annexin A2 immunostaining was also noted in the cancer associated stroma (Fig. 1D). Strong annexin A2 immunostaining was observed in the peritoneal cells of the omentum (Fig. 1E) and in the peritoneal cells adjacent to ovarian cancer cells in the omentum (Fig. 1F). No staining was observed in the absence of the primary antibody (insert, Fig. 1A). Stromal annexin A2 immunostaining in the invasive serous ovarian carcinomas (stage I to IV) was significantly increased compared with normal ovaries, serous cystadenomas, or serous borderline tumors (*P* < 0.0001). No difference was observed between the intensity of the annexin A2 immunostaining in the cancer cells (*P* = 0.510) or percentage of annexin A2 positive cancer cells (*P* = 0.248) between the different patients groups (Supplementary Table 1). No difference in annexin A2 immunostaining was observed between primary tumor and matching omental metastasis tissues (Supplementary Table 2). However, in metastatic omental implants, a higher proportion of ovarian cancer cells immediately adjacent to the peritoneal cells were annexin A2 positive (8/9) compared to ovarian cancers cells at a greater distance from the peritoneum (4/9) (*P* = 0.046, Pearson Chi Square) (Supplementary Table 3).

### Expression of annexin A2 in human ovarian cancer and peritoneal cell lines

**Figure 2:**
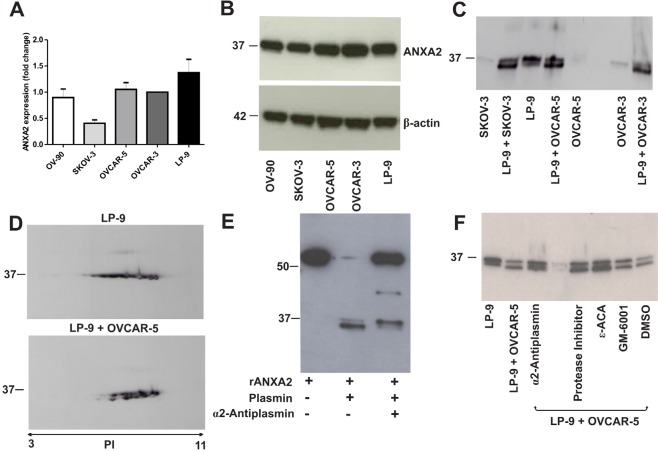
Annexin A2 expression in ovarian cancer cell lines, peritoneal cell line and co-cultured ovarian cancer and peritoneal cells (A) Annexin A2 expression in OV-90, SKOV-3, OVCAR-5, OVCAR-3 and LP-9 cell lines determined by real-time PCR and were assessed using 2^−ΔΔCT^ quantitation method. Data represents triplicate determinations ± SEM from 2 independent experiments. (B) Western immunoblotting shows annexin A2 band at 37 kDa in cell lysates of OV-90, SKOV-3, OVCAR-5, OVCAR-3 and LP-9. β-actin was used as loading control. (C) Western immunoblotting shows two isoforms of annexin A2 at 37 kDa and 36 kDa bands in the conditioned media (CM) of LP-9 cells alone and a 35 kDa annexin A2 band present in the CM of co-cultured LP-9 and ovarian cancer cells. (D) 2D-western immunoblotting showed multiple annexin A2 spots at 37 kDa in the CM of LP-9 cells alone and annexin A2 isoforms at 37 kDa and 35 kDa in the CM of co-cultured LP-9 and OVCAR-5 cells over a pI values range of 6 to 8. (E) Annexin A2 western immunoblotting of recombinant annexin A2 with GST tag (~63.4 kDa) and a cleaved annexin A2 at ~36 kDa in the presence of plasmin that was partially inhibited by α2-antiplasmin. (F) Annexin A2 western immunoblotting of the CM of co-cultured OVCAR-5 and LP-9 cells treated with α2-antiplasmin, protease inhibitor cocktail, ε-aminocaproic acid (ε-ACA), GM-6001 and DMSO.

Real-time PCR results showed annexin A2 was expressed in human serous ovarian cancer cell lines (OVCAR-3, OVCAR-5, SKOV-3 and OV-90) and the peritoneal cell line, LP-9 (Fig. 2A). This was supported by 1D-western immunoblotting in the cell lysates as an annexin A2 band at 37 kDa was observed in all cell lines (Fig. 2B). These findings confirm that annexin A2 is produced by both ovarian cancer cells and peritoneal cells.

### Annexin A2 is cleaved in ovarian cancer and peritoneal cells co-culture

Annexin A2 was found to be increased in the secretome of co-cultured OVCAR-5 and LP-9 cells using 2D gel electrophoresis and mass spectrometry [3]. Annexin A2 expression was further examined by western immunoblotting in the conditioned media (CM) from OVCAR-3, OVCAR-5, and SKOV-3 cells either cultured alone or in co-culture experiments with LP-9 cells after 48 h. A 37 kDa annexin A2 band at low abundance was observed in the CM from OVCAR-3, OVCAR-5, and SKOV-3 cells. In the LP-9 CM, two distinct bands corresponding to annexin A2 at approximately 37 and 36 kDa were observed. A shift in the 37 kDa band to a 35 kDa form was observed when LP-9 cells were co-cultured with OVCAR-3, OVCAR-5 or SKOV3 cells (Fig. 2C). A similar finding was observed with OV-90 and LP-9 cells (data not shown). Mass spectrometry of annexin A2 from the CM of the co-cultured OVCAR-5 and LP-9 (n=4) did not identify any annexin A2 peptides in the N-terminal domain (Supplementary Fig. 1). 2D-western immunoblotting was performed to confirm the presence of processed or cleaved annexin A2 in the co-cultured LP-9 and OVCAR-5 CM compared with LP-9 CM cultured alone (Fig. 2D). Multiple annexin A2 spots at 37 kDa and 35 kDa and pI values ranging from 6 to 8 in the co-cultured OVCAR-5 and LP-9 CM compared with the CM of LP-9 cells alone were observed. This result is consistent with the annexin A2 processing observed in the 1D-western immunoblotting.

### Annexin A2 is cleaved by protease plasmin

Plasmin digestion experiment demonstrated that recombinant annexin A2 is cleaved by plasmin and annexin A2 cleavage was partially inhibited following treatment with α2-antiplasmin (Fig. 2E). To evaluate whether annexin A2 processing was mediated by other proteases, co-cultured OVCAR-5 and LP-9 cells were treated with MMP inhibitor, GM-6001, a broad spectrum protease inhibitor cocktail as well as plasmin inhibitors, ε-ACA and α2-antiplasmin. In the co-culture experiment, only α2-antiplasmin was able to partially block annexin A2 cleavage (Fig. 2F). The other inhibitors used in the co-culture experiment had no effect on annexin A2 processing.

### Annexin A2 promotes ovarian cancer motility and invasion and aids peritoneal adhesion of ovarian cancer cells

**Figure 3:**
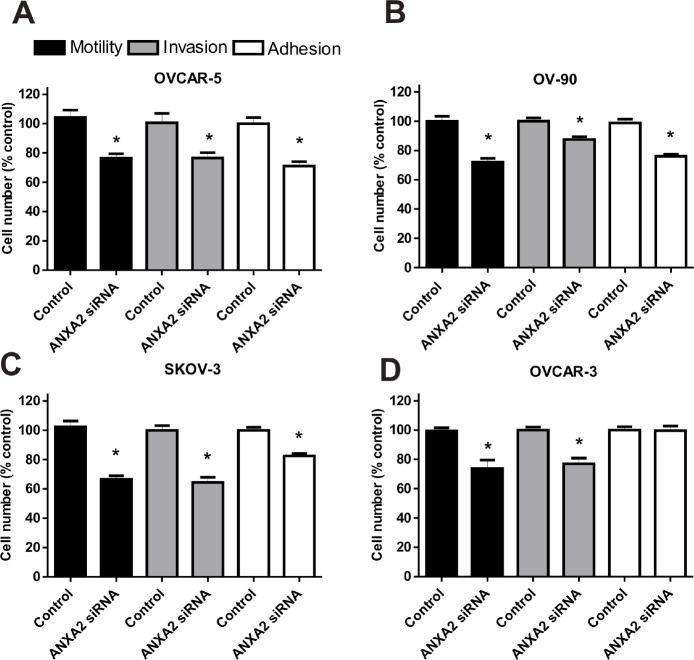
The effects of annexin A2 siRNA knockdown on ovarian cancer cell motility, invasion and adhesion *in vitro* (A) OVCAR-5, (B) OV-90, (C) SKOV-3 and (D) OVCAR-3 cell motility, invasion and adhesion to the peritoneal cells after treatment with annexin A2 siRNA. Data are expressed as a percentage of the negative control siRNA, mean ± SEM of quadruplicates from 3 independent experiments (n=12). *, significantly different from control (*P* < 0.05, Student *t*- test).

**Figure 4:**
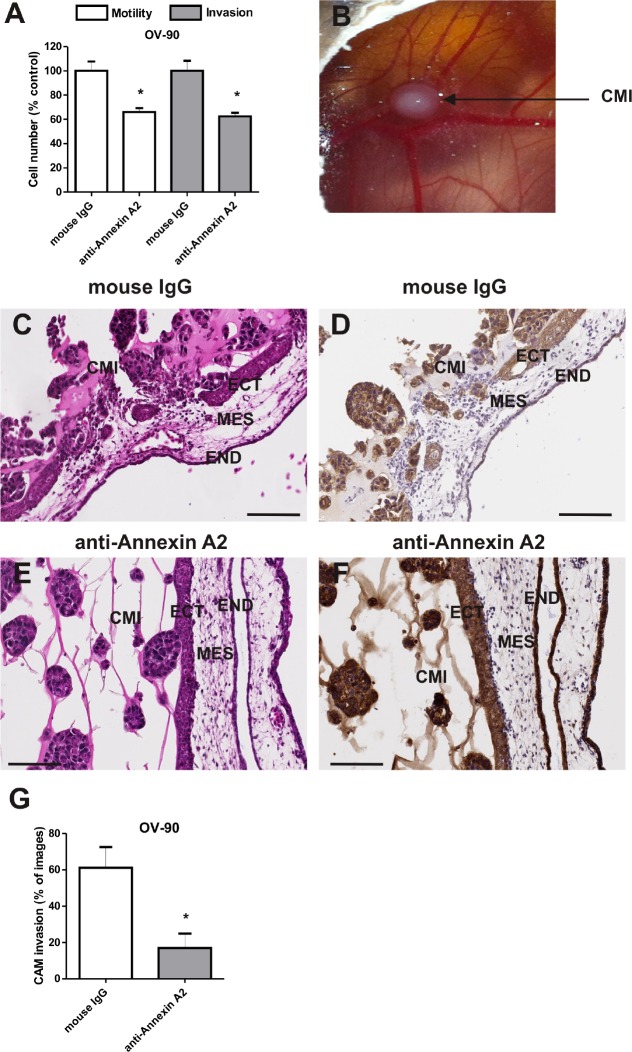
Annexin A2 promotes OV-90 cell motility and invasion *in vitro* and *in vivo* (A) Cell motility and invasion of OV-90 cells treated with mouse IgG antibody or annexin A2 neutralizing antibody *in vitro*. Data are expressed as a percentage of control mouse IgG, mean ± SEM of quadruplicates from 3 independent experiments (n=12). *, significantly different from control (*P* < 0.05, Student *t*-test). (B) Chick chorioallantoic membrane (CAM) implanted with matrigel and OV-90 cancer cells (CMI) at day 14 of chick embryo development. The CAM layers ectoderm (ECT), mesoderm (MES) and endoderm (END) are evident. Haematoxylin and eosin and pan-cytokeratin immunohistochemistry of the CAM implanted with OV-90 cells in the presence of mouse IgG antibody (C,D) and annexin A2 neutralizing antibody (E,F). (G) Quantitative analysis of OV-90 cells invasion into the CAM. Data represents mean ± SEM from 72-80 images (n=12 chick embryos per treatment group from 2 independent experiments). *, significantly different from control (*P* < 0.05, Mann-Whitney U test). Magnification bar = 100 μm for all images.

To determine whether annexin A2 promotes ovarian cancer metastasis, we examined the effects of knocking down annexin A2 expression on ovarian cancer cell adhesion to the peritoneal cells, motility, and invasion. Real-time PCR results showed annexin A2 siRNAs effectively knocked down annexin A2 expression up to 70% compared with negative control siRNA and non-treated OVCAR-5 cells (Supplementary Fig. 2A). Moreover, the knockdown of annexin A2 expression with siRNA A into OVCAR-3, OVCAR-5, SKOV-3, and OV-90 cells were confirmed by western immunoblotting (Supplementary Fig. 2B). Annexin A2 siRNA treated OVCAR-5, OV-90, SKOV-3, and OVCAR-3 cells showed significantly decreased motility (OVCAR-5 (*P* = 0.0008), OV-90 (*P* < 0.0001), SKOV-3 (*P* < 0.0001) and OVCAR-3 (*P* = 0.0069) and invasion (OVCAR-5 (*P* = 0.0047), OV-90 (*P* = 0.0047), SKOV-3 (*P* < 0.0001) and OVCAR-3 (*P* = 0.0002), compared with cells treated with the negative control siRNA (Fig. 3A-D). Furthermore, annexin A2 siRNA treated SKOV-3, OVCAR-5, and OV-90 cells had significantly decreased adhesion to LP-9 cells when compared with cells treated with negative control siRNA (Fig. 3A-C, OVCAR-5 (*P* = 0.0005), OV-90 (*P* < 0.0001), SKOV-3 (*P* < 0.0001)). However, there was no difference in the adhesion to the peritoneal cells in annexin A2 siRNA treated OVCAR-3 cells (Fig. 3D, *P* = 0.9057). We also observed a significant decrease in OV-90 cell motility (*P* = 0.0016) and invasion (*P* = 0.0031) after treatment with annexin A2 neutralizing antibody, compared with the mouse IgG antibody (Fig. 4A). Treatment of OVCAR-5 cells with annexin A2 neutralizing antibody also showed a significant decrease in cell motility and invasion compared with the mouse IgG antibody (data not shown).

### Annexin A2 promotes ovarian cancer cell invasion in the chick chorioallantoic membrane model

Ovarian cancer cell invasion was assessed using the CAM (chick chorioallantoic membrane) assay. OV-90 cells were mixed together with matrigel and placed onto the CAM of the chick embryos (Fig. 4B). To evaluate the effects of annexin A2 on ovarian cancer cell invasion *in vivo*, OV-90 cells were treated with the mouse IgG antibody and annexin A2 neutralizing antibody. Haematoxylin and eosin staining and pan-cytokeratin immunostaining of OV-90 cells treated with the mouse IgG antibody showed invasion of OV-90 cancer cells through the ectoderm into the mesoderm of the CAM and a destruction of the ectoderm layer (Fig. 4C,D). In contrast, OV-90 cells treated with annexin A2 neutralizing antibody exhibited minimal invasion through the ectoderm and mesoderm of the CAM (Fig. 4E,F). Quantitative analysis showed that annexin A2 antibody significantly inhibited OV-90 cancer cell invasion into the CAM mesoderm. Treatment with annexin A2 antibody resulted in a 3.6 fold reduction in cancer cell invasion, compared with OV-90 cancer cells treated with mouse IgG antibody (Fig. 4G, *P* = 0.004, Mann-Whitney U test).

### Annexin A2 promotes ovarian cancer growth and metastasis

**Figure 5:**
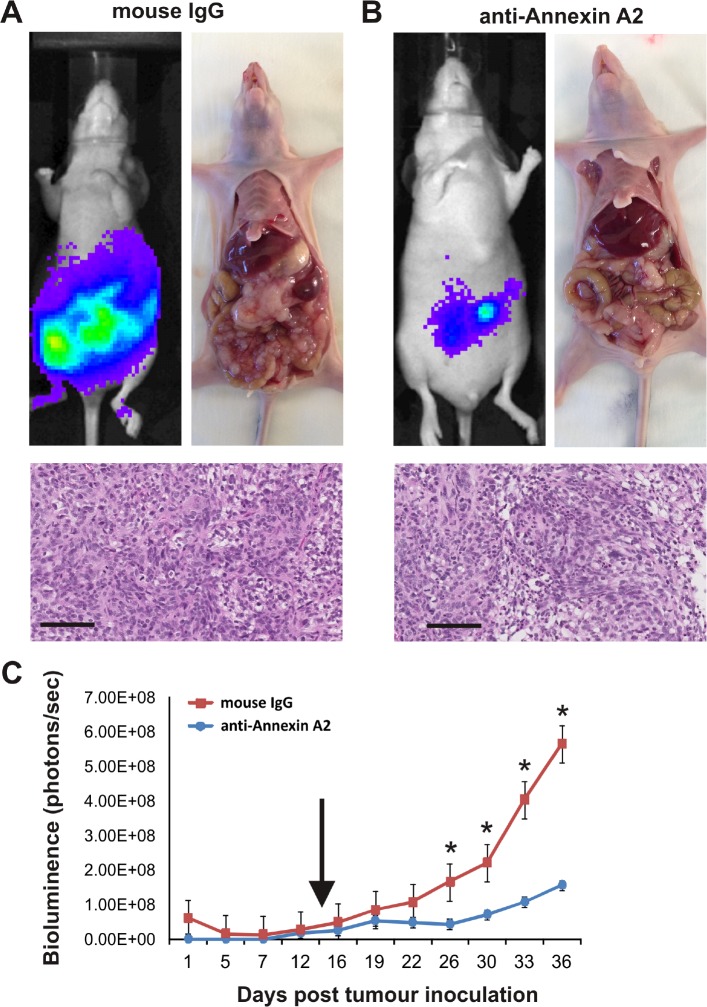
Annexin A2 promotes growth and metastasis of SKOV-3 cells Tumor burden of mice treated with mouse IgG antibody (A) or anti-annexin A2 antibody (B) on day 36 using IVIS imaging and at autopsy, with haematoxylin and eosin staining showing the tumor morphology. Images represent 1 sec acquisition time, and the photon emission transmitted from mice was captured and quantitated in photons/s/cm^2^/sr. (C) Tumor growth and metastasis measured by bioluminescence. Arrow indicates the time point of weekly antibody administration until day 36 (*P < 0.05, Mann-Whitney U test). Data represents mean ±SEM (n=5 mice per treatment group). Magnification bar = 100 μm for all images.

We utilized an intraperitoneal xenograft mouse model to assess the role of annexin A2 in ovarian cancer metastasis *in vivo*. The extensive tumor development and peritoneal metastasis observed with mouse IgG treatment (Fig. 5A) was significantly reduced in mice treated with annexin A2 neutralizing antibody (Fig. 5B). Tumor burden measured by bioluminescence was significantly reduced by annexin A2 neutralizing antibody treatment compared with mouse IgG antibody treatment over a 36 day period (Fig. 5C, Supplementary Fig. 3 and *P* < 0.05, Mann-Whitney U test).

**Figure 6:**
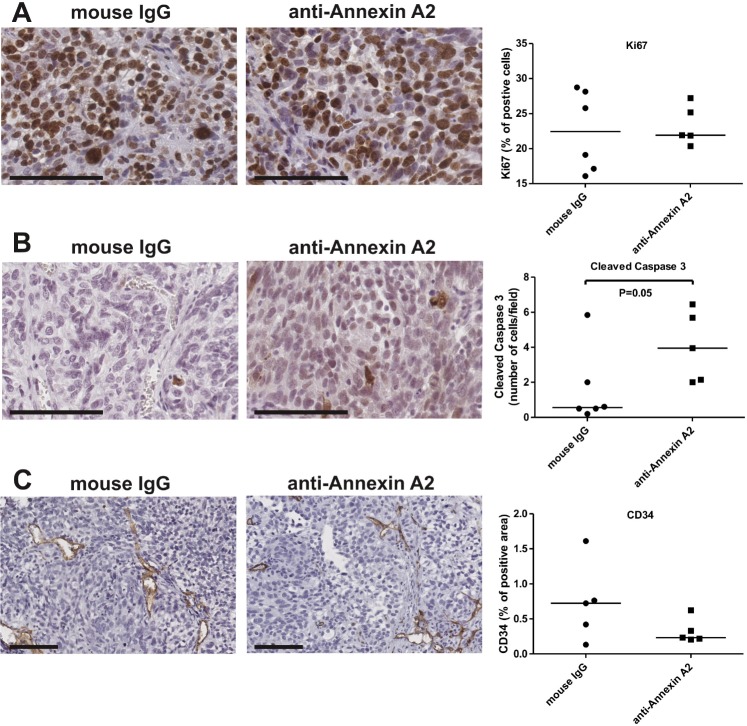
Annexin A2 neutralizing antibodies increase apoptosis Tumor sections of mice treated with mouse IgG and anti-annexin A2 antibodies were immunostained with (A) Ki67, (B) cleaved caspase 3 and (C) CD34. Data represents the median from 10 images per mice (n=6 for mouse IgG and n=5 for anti-annexin A2 treatment groups). *, significantly different from control (*P* < 0.05, Mann-Whitney U test). Magnification bar = 100 μm for all images.

We investigated the possible mechanisms for the reduced tumor burden in the annexin A2 antibody treatment group by analysing tumor cell proliferation, cell apoptosis and angiogenesis. Neutralizing annexin A2 antibody had no effect on SKOV-3 cell proliferation (Ki67, Fig. 6A), but significantly increased SKOV-3 cell apoptosis (active caspase 3, Fig. 6B). No difference in vascular density was observed between the two treatment groups (CD34, Fig. 6C). Our findings suggest the reduced tumor burden and metastatic spread in the annexin A2 antibody treatment group is a result of reduced cell survival.

## DISCUSSION

To our knowledge, this is the first study to investigate the role of annexin A2 in ovarian cancer invasion and metastasis. Here we show i) high annexin A2 expression in 90% of serous ovarian cells and increased annexin A2 levels in the cancer-associated stroma, ii) suppression of annexin A2 expression significantly reduces migration and invasion of 4 ovarian cancer cell lines, iii) down regulation of annexin A2 reduces adhesion of ovarian cancer cells to the peritoneal cells, and iv) annexin A2 neutralizing antibodies blocks migration and invasion of ovarian cancer cells, both *in vitro* and *in vivo*. Collectively these results provide strong evidence that annexin A2 plays a pivotal role in ovarian cancer progression and metastasis.

Ovarian cancer cell implantation onto the peritoneal lining is a vital step in ovarian cancer metastasis. However, the mechanisms involved in this process are poorly characterized. In this study, annexin A2 was regulated as a result of ovarian cancer and peritoneal cell interactions. Although annexin A2 over expression has been observed in various cancer types such as those of the pancreas [16], colorectal [17], breast [11, 18], prostate [13], liver [19] and brain [9, 20], to date, no information on the expression of annexin A2 in human ovarian cancer tissues and its functional role in ovarian cancer have been published.

Our immunohistochemical studies showed annexin A2 expression in both the membrane and cytoplasm of ovarian cancer cells, but high annexin A2 levels were also observed in the cancer associated stroma. Strong annexin A2 expression in stromal cells was observed for all clinical stages of human ovarian cancer (stage I to IV) compared with normal ovaries, serous cystadenomas and borderline ovarian tumors. Increased levels of annexin A2 were also present in ovarian cancer cells located adjacent to the peritoneal cells in the omental implants tissues.

Annexin A2 was shown to be expressed in human ovarian cancer and peritoneal cell lines. We observed a shift in the full length 37 kDa annexin A2 band to a 35 kDa isoform in the CM of co-cultured ovarian cancer and peritoneal cells. Furthermore, peptide mass fingerprinting of the annexin A2 protein in the co-cultured CM failed to detect any peptides containing the first 30 amino acids of the N-terminal domain of annexin A2. These findings suggest that annexin A2 is cleaved at the N-terminal domain as a result of the ovarian cancer-peritoneal cell co-culture environment.

We investigated whether annexin A2 cleavage in the ovarian cancer peritoneal cell co-culture could be inhibited by specific proteases. Plasmin is one of the proteases at the cell surface involved in remodeling the tumor microenvironment [21]. We previously showed that plasmin was upregulated in the CM of co-cultured ovarian cancer and peritoneal cells [2]. Previous studies reported annexin A2 cleavage by plasmin at lysine 27 in the N-terminal domain in monocytes [22], and between lysine 307 and arginine 308 in the C-terminal domain in endothelial cells [23]. Annexin A2 is also cleaved by proteases such as glycogen synthase-3 [24] and MMP-7 [25]. We confirmed that plasmin cleaved recombinant annexin A2 and we demonstrated that annexin A2 cleavage in co-cultured ovarian cancer and peritoneal cells could be partially blocked by α2-antiplasmin.

Our findings suggest that the extracellular form of annexin A2 found in the cancer associated stroma in the ovarian cancer tissues may represent a cleaved secreted form of annexin A2, which may assist ovarian cancer progression and metastasis. Since annexin A2 lacks a signal peptide and is not secreted via the endoplasmic reticulum pathway, the mechanism that regulates annexin A2 secretion remains unknown. It has been reported previously that cleavage of annexin A2 at lysine 10 by MMP-7 can assist tumor invasion and metastasis of colorectal and breast cancer cell lines [25]. Moreover, annexin A2 isoforms and cleaved annexin A2 were observed in normal and squamous cell carcinoma tissues, confirmed by 2D-western immunoblotting [26]. However, further studies are required to investigate annexin A2 cleavage mechanisms, the functional role of cleaved annexin A2 and post-translational modifications of annexin A2 in ovarian cancer.

In this study, we have demonstrated that suppression of annexin A2 using siRNA decreased ovarian cancer cell adhesion to the peritoneal cells, cell motility, and invasion *in vitro*. These observations concur with previous studies demonstrating decreased cell motility and invasion in cancers of the pancreas [10, 27], breast [28], brain [9], liver [12] and prostate [29] following treatment with annexin A2 siRNA. Moreover, our study demonstrated annexin A2 plays a role in ovarian cancer cell adhesion to the peritoneal cells. Shiozawa *et al.* reported that annexin A2 has a role in regulating prostate cancer cell adhesion to osteoblasts and endothelial cells using annexin A2 siRNA [13]. Silencing of annexin A2 expression significantly reduced cell adhesion of hepatocellular carcinoma cells [12] and cell adhesion of myeloid cells to human and murine osteoblasts cells [30]. Braden *et al.* also reported that down-regulation of annexin A2 expression using polymeric nanoparticles in prostate cancer cell line inhibits tumor growth in nude mice [29]. Therefore, annexin A2 contributes to cancer progression by enhancing cancer cell motility, invasion and adhesion.

The functional role of annexin A2 in ovarian cancer cell motility and invasion was also assessed using annexin A2 neutralizing antibody. We showed OV-90 cells treated with neutralizing annexin A2 antibody significantly decreased motility and invasion *in vitro* and invasion *in vivo* in the chick embryo CAM model. Annexin A2 neutralizing antibodies also significantly inhibited growth and metastasis of SKOV-3 cells in nude mice. Our finding are consistent with previous reports that have shown annexin A2 neutralizing antibodies to inhibit cell migration, invasion, and to block plasminogen activation of breast cancer cells [11] and monocytes [31] *in vitro*. Moreover, anti-annexin A2 antibodies have been shown to inhibit pancreatic cancer metastasis in a mouse model of pancreatic ductal adenocarcinoma [10] and to inhibit tumor growth in breast cancer and Lewis Lung Carcinoma xenograft mouse models [32, 33]. A significant reduction of glioma tumor growth and progression in the annexin A2 knockout mice model associated with a decrease in cancer cell invasion, angiogenesis and proliferation has also been reported [9]. Tumor growth of fibrosarcoma (HT1080) and lung cancer (A459) cell lines in NOD-SCID mice has also been shown to be inhibited following annexin A2 depletion [6]. Furthermore, Zhai *et al.* showed a decrease in glioma tumor burden as a result of an increase in apoptosis in tumors of the annexin A2 knockout mice [9]. Similarly, we observed an increase in cell apoptosis, but no difference in the cell proliferation or vascular density in the tumor sections of mice with SKOV-3/GFP-Luc cells treated with anti-annexin A2 antibody. The *in vivo* findings from our CAM model and intraperitoneal xenograft mouse model were consistent with our *in vitro* observations demonstrating annexin A2 promotes ovarian cancer cell motility, invasion and tumor growth.

In conclusion, our findings demonstrate that annexin A2 plays an important role in ovarian cancer metastasis. Anti-annexin A2 antibodies significantly blocked ovarian cancer cell invasion in the CAM model and cancer cell peritoneal dissemination in the intraperitoneal xenograft mouse model. Annexin A2 is therefore a promising novel therapeutic target against ovarian cancer.

## MATERIALS AND METHODS

### Patient's tissue samples

Archived formalin fixed paraffin tissue blocks from 20 matching primary tumors and their metastatic implants, 10 cases each of early stage serous ovarian cancer (stage I and II), advanced stage serous ovarian cancer (stage III and IV) and borderline ovarian tumors, 16 normal ovaries, 11 serous cystadenomas and 9 omental implants were obtained from the Institute of Medical Veterinary Science (SA Pathology), Adelaide, South Australia, Australia. Tissue microarrays (TMAs) were constructed from formalin-fixed, paraffin embedded tumor material with the approval from the Royal Adelaide Hospital ethics committee and each tissue block was represented by triplicate 1.0 mm diameter tissue cores.

### Cell culture

The human serous ovarian cancer cell lines OVCAR-3, SKOV-3 and OV-90 were purchased from American Type Culture Collection (ATCC, VA, USA). OVCAR-5 cells were obtained from Dr Thomas Hamilton (Fox Chase Cancer Center, PA, USA) and the peritoneal cells, LP-9 were purchased from Coriell Cell Repositories (NJ, USA). All cell lines were maintained as previously described [2]. The SKOV-3/GFP-Luc cells (Cell Biolabs Inc., CA, USA) were maintained in RPMI 1640 medium supplemented with 4mM L-glutamine, antibiotics (100 U penicillin G, 100 μg/ml streptomycin sulfate and 0.25 μg/ml amphotericin B) and supplemented with 5% fetal bovine serum (FBS) (Sigma-Aldrich, MO, USA). All cell lines were maintained at 37oC in an environment of 5% CO_2_.

### Real-time PCR

Total RNA was extracted using TRIzol (Invitrogen, NSW, Australia) and each RNA sample was reverse transcribed using SuperScript™ III Reverse Transcriptase (Invitrogen), as per manufacturer's instructions. Real-time PCR was performed in triplicates using human annexin A2 validated primers (QIAGEN, Australia) and SYBR Green PCR master mix (7900HT Fast Real-Time PCR System, Applied Biosystems, NSW, Australia). The cycling parameters were: 50oC for 2 min, 95oC for 10 min followed by 40 cycles of 95oC for 15 sec and then 60oC for 1 min. The CT values were normalised relative to the housekeeping gene, L19 and were calibrated to the OVCAR-3 cells using the 2^−(ΔΔCT)^ quantitation method.

### Immunohistochemistry

For the annexin A2 immunohistochemistry, tissue sections (5 μm) underwent microwave antigen retrieval (5 min 750 W, 15 min 350 W) in 10 mM citric acid buffer (pH 6.5), and were incubated overnight with mouse monoclonal antibody to annexin A2 (1/500, BD Biosciences, CA, USA) in blocking buffer (5% normal goat serum) at 4°C. Visualization of immunoreactivity was achieved using biotinylated anti-mouse immunoglobulins (1/400, Dako, NSW, Australia), streptavidin-peroxidise conjugate (1/500, Dako) and diaminobenzidine tetrahydrochloride (DAB) (Sigma-Aldrich), as previously described [2]. Normal prostate tissue was used as a positive control [34] and negative controls included no primary antibody and mouse IgG controls. For the xenograft mouse model experiment, mouse tumor tissue sections were immunostained with active caspase-3 (rabbit polyclonal, 1/200, Cell Signaling Technology, MA, USA), Ki67 (rabbit monoclonal, 1/400, Epitomics, CA, USA) and CD34 (rat monoclonal, 1/100, clone MEC 14.7, Abcam, MA, USA), as previously described [35].

### Co-culture of ovarian cancer and peritoneal cells

LP-9 cells were cultured in 6 well plates until they reached confluency. OVCAR-5, SKOV-3, OV-90 and OVCAR-3 cells were added (2 × 10^5^ cells/well) to the LP-9 monolayer and co-cultured in direct contact for 48 h before the collection of the cell lysates and CM, as previously described [2]. OVCAR-5 and LP-9 cells were also co-cultured in the presence or absence of protease inhibitor cocktail (1/200, Sigma-Aldrich), MMP inhibitor, GM-6001 (20 μM, Calbiochem, CA, USA) as well as plasmin inhibitors, ε-ACA (150 mM, Sigma-Aldrich) and α2-antiplasmin (0.2 μM, Calbiochem).

### 1D-western immunoblotting

Western immunoblotting was conducted as previously described [2] with a mouse monoclonal antibody to annexin A2 (1/2000, BD Biosciences) or β-actin antibody (1/2000, Abcam) and anti-mouse IgG peroxidase-conjugated secondary antibodies (1/2000, Dako). For the plasmin digestion, recombinant annexin A2 protein with a GST-tag (0.5 μg, Abnova, Taiwan) was incubated with plasmin (0.2 U/ml, Sigma-Aldrich) for 3 h at 37oC in the presence and absence of α2-antiplasmin (0.2 μM, Calbiochem).

### 2D-western immunoblotting

The total protein concentration of CM from LP-9 alone and co-cultured OVCAR-5 and LP-9 was determined using EZQ protein assay (Invitrogen). The immobilized pH gradient (IPG) strips (11cm, GE Healthcare, NJ, USA) were rehydrated in rehydration buffer containing 1.2% DeStreak, 0.5% C.A, 1% bromophenol blue and 4% thiourea-urea-CHAPS (TUC) buffer overnight in IPGPhorII (GE Healthcare). A total of 50 μg protein sample was reduced with 1 M DTT, 0.8% IPG buffer, TUC buffer and bromophenol blue and applied to the IPG strip and isoelectric focusing was carried out using IPGPhorII at 20°C as previously described [2]. Western immunoblotting was performed as per 1D-western immunoblotting.

### Motility and invasion assays

Cell motility and invasion assays were performed as previously described [36]. *For the annexin A2 knockdown experiments, ovarian cancer c*ells were transfected with annexin A2 siRNAs (siRNA ID: s1384 (A) and s1385 (B), 10 nM) or negative siRNA (Ambion, TX, USA) with Oligofectamine (Invitrogen) for 48 h. Ovarian cancer cells were also pre-treated with *annexin A2 neutralizing antibody (20 μg/ml*, BD Biosciences) or mouse IgG antibody (20 *μg/ml, Sigma-Aldrich) for 2 h*. Cells were *labelled with calcein-AM (1 μg/ml, Invitrogen) and were loaded onto* uncoated 12 μm filter inserts (96-well plate, ChemoTx, Neuro Probe, MD, USA) for migration assays or 12 μm filters coated with Geltrex (0.6 μl/well, Invitrogen) for invasion assays. The cells were allowed to migrate and invade to the lower chamber for 6 h and the *bottom* fluorescence of migratory cells was measured at 485-520 nm using the Triad series multimode detector (Dynex Technologies, VA, USA).

### Adhesion assay

Adhesion assay were performed as previously described [2]. Briefly, ovarian cancer cells were transfected with either annexin A2 siRNA A or negative siRNA for 48 h and labelled with calcein-AM. Ovarian cancer cells were added to the LP-9 cell monolayer and were allowed to adhere for 8 min. The fluorescence of adhered cells was measured at 485-520 nm using the Triad series multimode detector (Dynex Technologies).

### Chick chorioallantoic membrane assay

Fertilized white leghorn chicken eggs (Hi Chick, SA, Australia) were maintained at 37°C in 60% relative humidity in a Multiquip Incubator E2 (Multiquip Pty. Ltd., NSW Australia). Approval was obtained by the University of Adelaide Animal Ethics Committee. On day 3 of chick embryo development, a small opening was made under aseptic conditions in the egg shell. To investigate the effects of annexin A2 on ovarian cancer cell invasion in the CAM model, OV-90 cells (9×10^4^ cells) were mixed with matrigel (8.9 mg/mL, BD Biosciences) with either annexin A2 neutralizing antibody (20 μg/ml, BD Biosciences) or mouse IgG antibody (20 μg/ml, Sigma-Aldrich) in a total volume of 30 μl and placed on the CAM of day 11 chick embryos (n=6 chick embryos per treatment group). The invasion of the cancer cells through the ectoderm into the mesoderm was assessed on day 14 of chick embryo development in paraffin-embedded CAM sections stained with haematoxylin, eosin and pan-cytokeratin immunohistochemistry, as previously described [37].

### Intraperitoneal SKOV-3 xenograft mouse model

An intraperitoneal SKOV-3 xenograft non-invasive and whole-body bioluminescent imaging model was used with the approval obtained by the University of Adelaide Animal Ethics Committee. Nude mice were injected intraperitoneally with SKOV-3/GFP-Luc cells (1×10^8^ cells/0.5ml) and were treated weekly with either 100 μg mouse IgG antibody or 100 μg anti-annexin A2 antibodies (BD Biosciences). Mice were injected intraperitoneally with D-luciferin (Caliper Life Sciences, MA, USA) solution at 150 mg luciferin/kg body weight and then gas-anesthetized with isoflurane (Bomac Pty. Ltd., NSW, Australia). Precisely 10,15 and 20 min following D-luciferin injection, images were acquired for 0.5 to 10 sec and the photon emission transmitted from mice was captured and quantitated in photons/s/cm^2^/sr using the IVIS Imaging System 100 (Xenogen Imaging Technology, CA, USA) with living image software (Igor Pro version 2.5). At autopsy, tumor tissues were fixed with 4% paraformaldehyde (Sigma-Aldrich) and embedded into paraffin blocks.

### Immunohistochemical assessment

Slides were digitally scanned using the NanoZoomer (Hamamatsu Photonics, Japan) and images were captured using NDP view imaging software (Hamamatsu Photonics). The immunostaining intensity of annexin A2 in the epithelial and stromal compartments was assessed using a manual scoring method: strong (3+), moderate (2+), weak (1+), or negative (0). A score of 0 or 1+ was defined as low annexin A2 immunostaining and a score or 2+ or 3+ was defined as high annexin A2 immunostaining. The percentage of annexin A2 positive cancer cells were independently assessed in the ovarian cancer tissues. For the CAM assay, quantitative analysis to assess OV-90 cancer cell invasion was performed on 8 to 12 CAM images for each embryo as previously described [37]. For the mouse tumor tissue sections, ten random images of each tissue were captured at 40x magnification for Ki67 and activated caspase-3 and at 20x magnification for CD34. Colour threshold detection by the AnalySIS-Pro™ software (Soft Imaging System, Germany) was used to determine positive (brown pixels) and negative (purple pixels) stained cells. Data was expressed as percentage of positive pixels (positive brown stained divided by negative purple stained area) for Ki67 and percentage of positive CD34 pixels of the total tumor area. For active caspase 3, the number of positive cells in ten fields were counted manually and expressed as a percentage of the total tumor area. The mouse treatment groups were blinded until completion of all analyses.

### Statistical analysis

All statistical analyses were performed using SPSS 19.0 for Windows (SPSS Inc., IL, USA). The Chi-Square test was used to determine statistical significance between annexin A2 immunostaining in the ovarian and omental tissue groups. The Student's *t*-test and one-way ANOVA with Dunnett C or Dunnett T post-hoc tests were used to determine statistical significance between control and treatment groups. For the CAM assay and xenograft mouse model experiments, the Mann-Whitney U test was used to determine the significance between control and treatment groups. Statistical significance was accepted at *P* < 0.05.

## Supplementary Tables and Figures




