# Supplementary material for: Annexin A2 is regulated by ovarian cancer-peritoneal cell interactions and promotes metastasis
Source: Oncotarget. 2013 Jul 14;4(8):1199–211. doi: 10.18632/oncotarget.1122 (PMC3787151; doi:10.18632/oncotarget.1122)
Supplement: Supplementary file 1 [file oncotarget-04-1199-s001.pdf]

Annexin A2 is regulated by ovarian cancer-peritoneal cell interactions and promotes metastasis – Lokman et al

Supplementary Table 1: Annexin A2 epithelial and stromal immunostaining intensity in the normal ovaries, serous cystadenomas, serous borderline tumors and serous ovarian carcinomas (stage I-IV) tissues.

Tissue	n	Patient age (years) Median (range)	Annexin A2 epithelial staining				Annexin A2 stromal staining	
			Intensity		% of positive cells		Intensity	
			Low[a]	High[b]	≤10	≥10	Low[a]	High[b]
Normal ovaries	16	52 (40-83)	2/16 (12.5%)	14/16 (87.5%)	1/16 (6.3%)	15/16 (93.8%)	14/16 (87.5%)	2/16 (12.5%)
Serous cysta denomas	11	50 (39-66)	4/11 (36.3%)	7/11 (63.6%)	2/11 (18.1%)	9/11 (81.8%)	10/11 (90.9%)	1/11 (9%)
Serous borderline tumors	10	50 (32-81)	0/10 (0%)	10/10 (100%)	0/10 (0%)	10/10 (100%)	8/10 (80%)	2/10 (20%)
Serous ovarian carcinomas (Stage I-IV)	42	64 (27-86)	4/42 (10%)	38/42 (90%)	9/42 (21.5%)	33/42 (78.5%)	14/42 (32.8%)	28/42 (67.3%)
Chi-squared test			$P = 0.510$		$P = 0.248$		$P < 0.0001$	

[a] Annexin A2 low intensity score; no staining (0) and weak staining (1+)
[b] Annexin A2 high intensity score; moderate staining (2+) and strong staining (3+)

Supplementary Table 2: Annexin A2 epithelial and stromal immunostaining intensity in the primary tumor and matching omental metastasis tissues.

Tissue	n	Patient age (years) Median (range)	Annexin A2 epithelial staining				Annexin A2 stromal staining	
			Intensity		% of positive cells		Intensity	
			Low[a]	High[b]	≤10	≥10	Low[a]	High[b]
Primary tumor	21	68 (49-87)	0/21 (0%)	21/21 (100%)	7/21 (33.3%)	14/21 (66.7%)	9/21 (42.9%)	12/21 (57.2%)
Matching omental metastasis	18		3/18 (16.7%)	15/18 (83.3%)	10/18 (55.6%)	8/18 (44.4%)	4/18 (22.2%)	14/18 (77.8%)
Fisher's exact test			*P* = 0.089		*P* = 0.206		*P* = 0.307	

[a] Annexin A2 low intensity score; no staining (0) and weak staining (1+)
[b] Annexin A2 high intensity score; moderate staining (2+) and strong staining (3+)

Supplementary Table 3: Annexin A2 epithelial immunostaining intensity in the omental tissues.

Omental Tissue	n	Patient age (years) Median (range)	Annexin A2 epithelial staining			
			Intensity		% of positive cells	
			Low[a]	High[b]	≤10	≥10
Cancer cells adjacent to the peritoneum	9	58 (40-70)	1/9 (11.1%)	8/9 (88.9%)	1/9 (11.1%)	8/9 (88.9%)
Cancer cells distant from the peritoneum	9	58 (40-70)	4/9 (44.4%)	5/9 (55.5%)	5/9 (55.5%)	4/9 (44.4%)
Chi-squared test			*P* = 0.114		*P* = 0.046	

[a] Annexin A2 low intensity score; no staining (0) and weak staining (1+)
[b] Annexin A2 high intensity score; moderate staining (2+) and strong staining (3+)
