# Supplementary material for: Annexin A2 is regulated by ovarian cancer-peritoneal cell interactions and promotes metastasis
Source: Oncotarget. 2013 Jul 14;4(8):1199–211. doi: 10.18632/oncotarget.1122 (PMC3787151; doi:10.18632/oncotarget.1122)
Supplement: Supplementary file 2 [file oncotarget-04-1199-s002.pdf]

Annexin A2 is regulated by ovarian cancer-peritoneal cell interactions and promotes metastasis – Lokman et al

```
1   MSTVHEILCK LSLEGDHSTP PSAYGSVKAY TNFDAERDAL NIETAIKTKG
51  VDEVTIVNIL TNRSNAQRQD IAFAYQRRTK KELASALKSA LSGHLETVIL
101 GLLKTPAQYD ASELKASMKG LGTDEDSLIE IICSRTNQEL QEINRVYKEM
151 YKTDLEKDII SDTSGDFRKL MVALAKGRRA EDGSVIDYEL IDQDARDLYD
201 AGVKRKGTDV PKWISIMTER SVPHLQKVFD RYKSYSPYDM LESIRKEVKG
251 DLENAFLNLV QCIQNKPLYF ADRLYDSMKG KGTRDKVLIR IMVSRSEVDM
301 LKIRSEFKRK YGKSLYYYIQ QDTKGDYQKA LLYLCGGDD
```

Supplementary Figure 1: Ion-trap mass spectrometry LC-MS/MS analysis of annexin A2 spots from the 2D gel electrophoresis. No annexin A2 peptides (red peptides: positive identified sequence via Mascot search) were observed in the N-terminal domain of annexin A2 (amino acid 1-30) in the protein spots of the 2D gel electrophoresis-silver stained gel from OVCAR-5 and LP-9 co-cultured cells conditioned media [3].

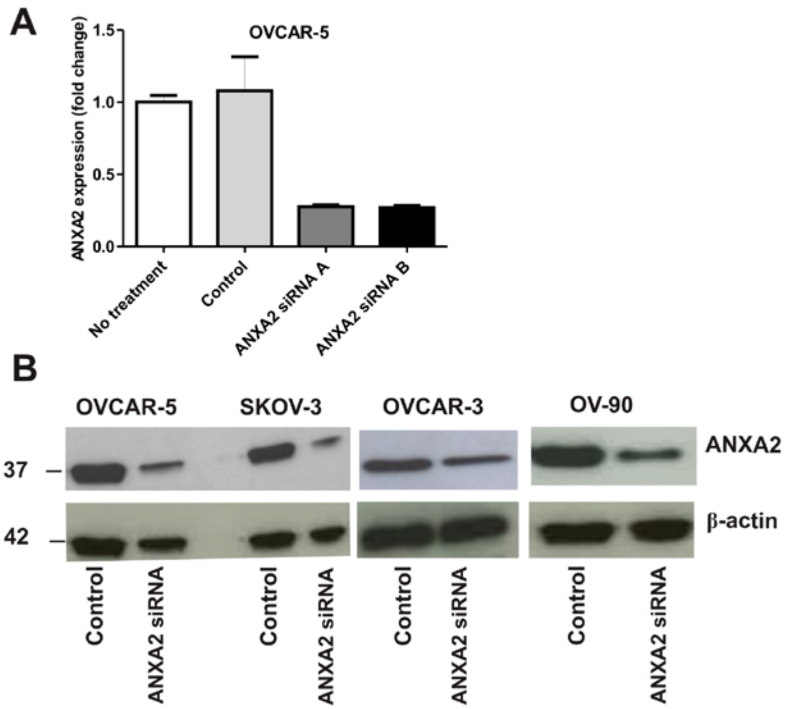

Supplementary Figure 2: Annexin A2 expression after knockdown with annexin A2 siRNAs. (A) Annexin A2 real-time PCR expression of OVCAR-5 cells after treatment with annexin A2 siRNA A and siRNA B compared with the negative control siRNA and non-treated cells, assessed using $2^{-\Delta\Delta CT}$ quantitation method. Data represents triplicate determinations ± SEM from 2 independent experiments. (B) Annexin A2 expression of OVCAR-5. SKOV-3, OVCAR-3 and OV-90 cells after knockdown with annexin A2 siRNA A and negative control siRNA confirmed by 1D-western immunoblotting. β-actin was used as a loading control.

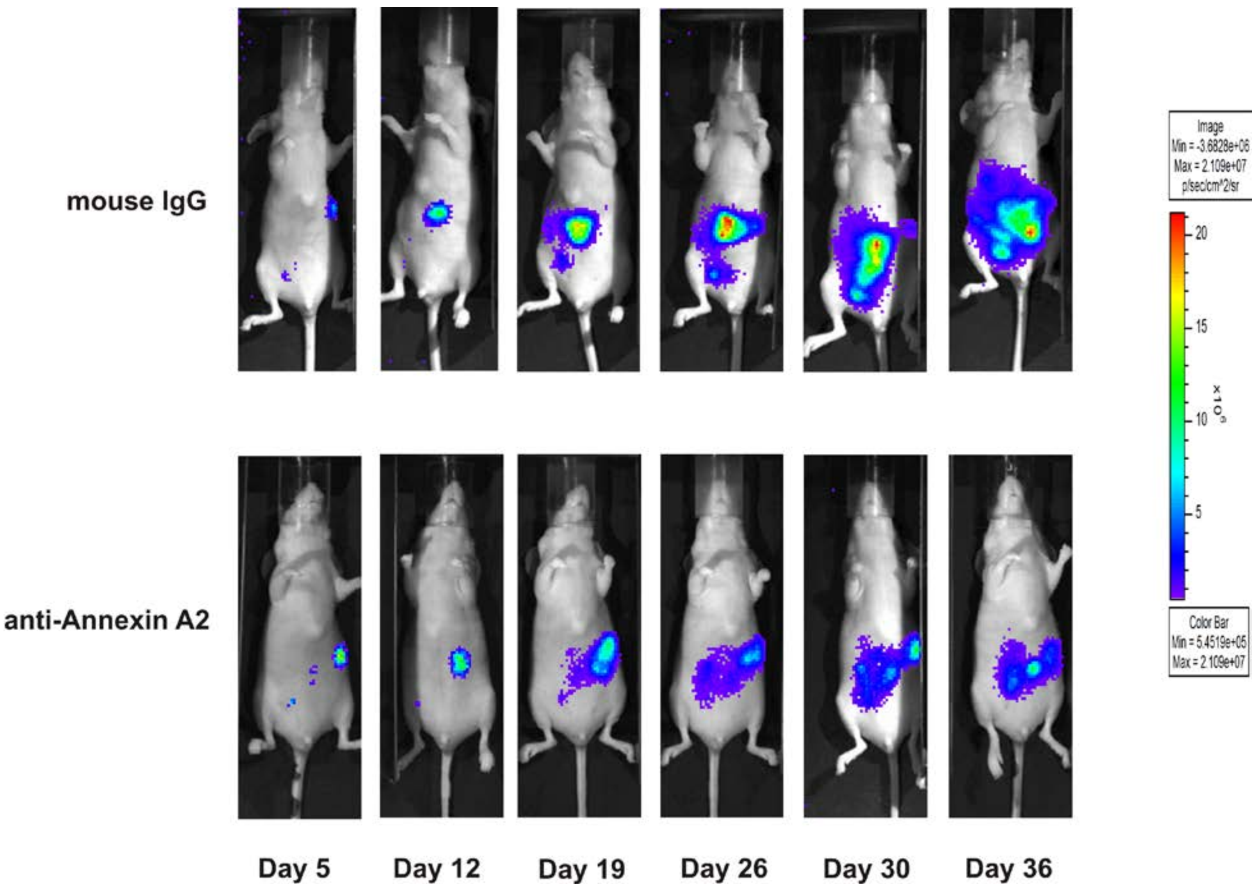

Supplementary Figure 3: IVIS imaging of mice. Mice were treated once weekly for three weeks with either mouse IgG (top row), or anti-annexin A2 antibody (bottom row). Images represent tumor growth on days 5, 12, 19, 26, 30 and 36, assessed using the IVIS Imaging. Images represent 1 sec acquisition time, and the photon emission transmitted from mice was captured and quantitated in photons/s/cm^2/sr.
